# A Direct Bicarbonate Detection Method Based on a Near-Concentric Cavity-Enhanced Raman Spectroscopy System

**DOI:** 10.3390/s17122784

**Published:** 2017-12-01

**Authors:** Dewang Yang, Jinjia Guo, Chunhao Liu, Qingsheng Liu, Ronger Zheng

**Affiliations:** College of Information Science & Engineering, Ocean University of China, Qingdao 266000, China; dewangyang@sdlaser.cn (D.Y.); liuchforever@163.com (C.L.); qingxing_liu@foxmail.com (Q.L.); rzheng@ouc.edu.cn (R.Z.)

**Keywords:** laser Raman spectroscopy, bicarbonate, direct detection, ocean application

## Abstract

Raman spectroscopy has great potential as a tool in a variety of hydrothermal science applications. However, its low sensitivity has limited its use in common sea areas. In this paper, we develop a near-concentric cavity-enhanced Raman spectroscopy system to directly detect bicarbonate in seawater for the first time. With the aid of this near-concentric cavity-enhanced Raman spectroscopy system, a significant enhancement in HCO_3_^−^ detection has been achieved. The obtained limit of detection (LOD) is determined to be 0.37 mmol/L—much lower than the typical concentration of HCO_3_^−^ in seawater. By introducing a specially developed data processing scheme, the weak HCO_3_^−^ signal is extracted from the strong sulfate signal background, hence a quantitative analysis with R^2^ of 0.951 is made possible. Based on the spectra taken from deep sea seawater sampling, the concentration of HCO_3_^−^ has been determined to be 1.91 mmol/L, with a relative error of 2.1% from the reported value (1.95 mmol/L) of seawater in the ocean. It is expected that the near-concentric cavity-enhanced Raman spectroscopy system could be developed and used for in-situ ocean observation in the near future.

## 1. Introduction

The carbon cycle in the ocean is a dynamic component of the global carbon budget, but the diverse sources and carbon sinks as well as their complex interactions in the ocean remain poorly understood [1,2]. The dissolved inorganic carbon (DIC) in the ocean is the sum of HCO_3_^−^, CO_3_^2−^, H_2_CO_3_ and CO_2_ [3]. The concentration of DIC is about 2.30 mmol/L, with ${\mathrm{HCO}}_{3}^{–}$ (about 1.95 mmol/L) making up the majority of it [4]. Therefore, bicarbonate is a key factor in carbon cycle research. The modern methods for oceanic carbonate system measurement are mainly involved in total carbon and CO_2_ partial pressure (pCO_2_) investigations [5,6]. However, the direct detection of HCO_3_^−^ and CO_3_^2−^ in seawater it is still a huge challenge due to their low concentrations. Raman spectroscopy is potentially capable of overcoming this challenge on the condition of a significant ensitivity enhancement [7].

In 2004, scientists reported the first attempt to detect HCO_3_^−^ in water solutions directly by using Raman spectroscopy [8], with a limit of detection (LOD) of 7.5 mmol/L after an integration time of 20 min. They had also tried to enhance the HCO_3_^−^ signal with an introduced liquid core waveguide [9], eventually obtaining an amplification ratio of 7.8× in the peak intensity. All those efforts still do not make the direct Raman detection of HCO_3_^−^ in seawater practical due to the fact its concentration is as low as 2 mmol/L. The strong background signal resulting from SO_4_^2−^, which has a concentration of ~28 mmol/L in seawater [10], makes direct detection of HCO_3_^−^ even more difficult. With a similar waveguide-based enhancement mechanism, the multi-pass cavity concept has been presented as an effective approach in gas Raman detection with desirable enhancement [11,12,13,14]. Taking such an approach in direct seawater Raman detection is the motivation for the work presented in this article. A near-concentric cavity-enhanced Raman spectroscopy system was specially built for liquid sample detection. With the aid of this system, direct HCO_3_^−^ Raman detection in seawater becomes readily feasible as expected.

## 2. Experiment Setup 

**Figure 1:**
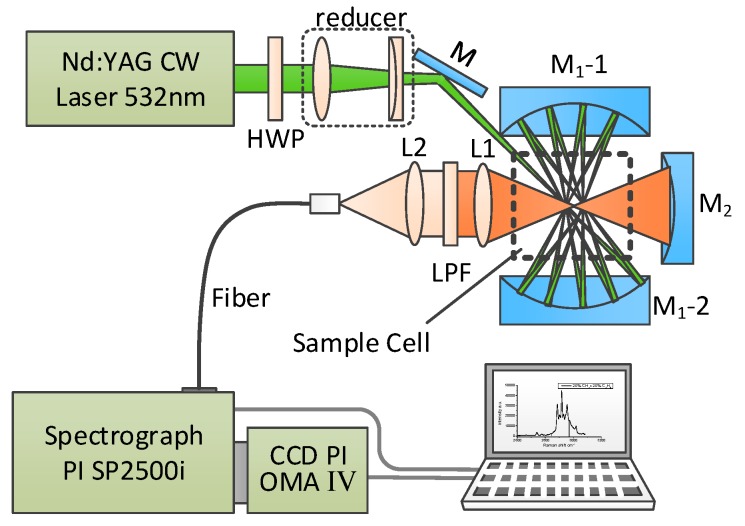
Schematic of the near-concentric cavity-enhanced Raman spectroscopy system for liquid sample detection.

As shown in Figure 1, the near-concentric cavity-enhanced Raman spectroscopy system is adapted on the basis of a formerly reported design [14]. In order to detect liquid samples, a sample cell of fused silica with 10 mm × 10 mm × 40 mm inner size and 1 mm wall thickness is used and placed at the center of the cavity. In this system, a diode-pumped, frequency-doubled Nd:YAG CW laser (532 nm) with a power of 300 mW is used as the Raman excitation laser source. The polarization of the laser beam is rotated by 90° through passing a half wave plate (HWP), and then the laser beam is compressed and focused into the chamber by a couple of lens (a planoconvex lens with a focal length of 100 mm and a planoconcave lens with a focal length of −75 mm). The near concentric cavity is composed of two identical spherical mirrors M1 with 25.4 mm diameter and 25 mm focal length. The reflectivity of these two mirrors is over 99% from 500 to 700 nm. These two mirrors are spaced face to face at a distance of 104.2 mm, and the mirror M1-2 is clockwise rotated about 0.04° to create the near concentric cavity reflection mode. Because of the strong absorption of water, the laser beam number in the cavity is 18, which is less than the number in the cavity of a gas sample cell. The scattering signal from the center of cavity is collected by an achromatic doublet lens L1 (f = 30 mm) with a diameter of 25.4 mm. A mirror M2 (f = 12.5 mm), placed on the opposite side beyond the sample chamber, is used to enhance the collection efficiency of the signal. Another lens L2 (f = 30 mm) is used to couple the Raman signal into the delivery optical fiber bundle (19 μm × 100 μm, NA = 0.22), with a long pass filter placed on its front to get rid of Rayleigh scattering from the collected signal. From the other end of the fiber, the signal is then coupled into a spectrometer (Acton SP2500i, Princeton Instruments, Trenton, NJ, USA), with 1200 g/mm grating and 10 μm entrance slit width. The Raman spectra are recorded by a CCD detector (SPEC-10: 400B, Princeton Instruments, Trenton, NJ, USA) with a 1340 × 400 imaging array and 20 μm × 20 μm pixel size operating at −40 °C. The resulted spectral range is 800~2000 cm^−1^ with a resolution of 2 cm^−1^. To get a relative desirable Raman signal and prevent the CCD from overexposure, each spectrum is accumulated five times with an integration time of 10 s, so the total acquisition time is 50 s for one sample. Five measurements are taken for each sample to evaluate the repeatability.

## 3. Results

**Figure 2:**
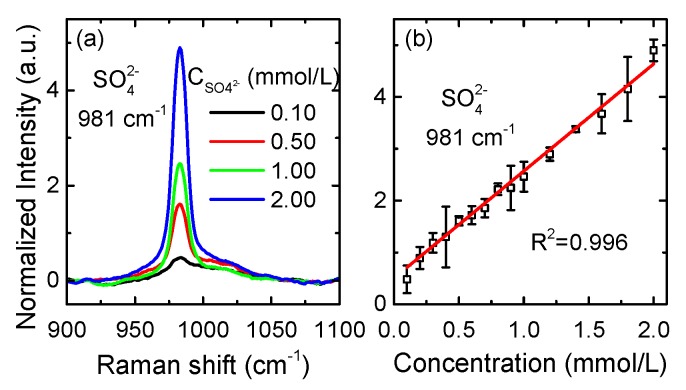
Raman spectra of a range of Na_2_SO_4_ solution (0.10 mmol/L to 2.00 mmol/L). (**a**) Four spectra of typical concentration. (**b**) The linear relationship between concentrations and peak intensities of the SO_4_^2−^ signal.

The reported LOD for SO_4_^2−^ by using a liquid core optical fiber Raman system is about 1.5 mmol/L [15]. As a comparison, we prepared 15 Na_2_SO_4_ solutions (0.10 mmol/L–2.00 mmol/L) to evaluate the LOD of the NC-CERS system. The LOD is defined as the ratio of three times the noise intensity (σ) to the slope of calculation curve. The spectra of four solutions are shown in Figure 2a, and the Raman signal of SO_4_^2−^ can be observed clearly in the spectrum of the 0.10 mmol/L solution. Given this, the NC-CERS system has a LOD less than 0.10 mmol/L for SO_4_^2−^ which is 15 times lower than that reported before. The linear relationships between concentrations and peak intensity of the SO_4_^2−^ are shown in Figure 2b and the response of the NC-CERS system for liquid detection has good linearity with R^2^ = 0.996 over the whole range.

**Figure 3:**
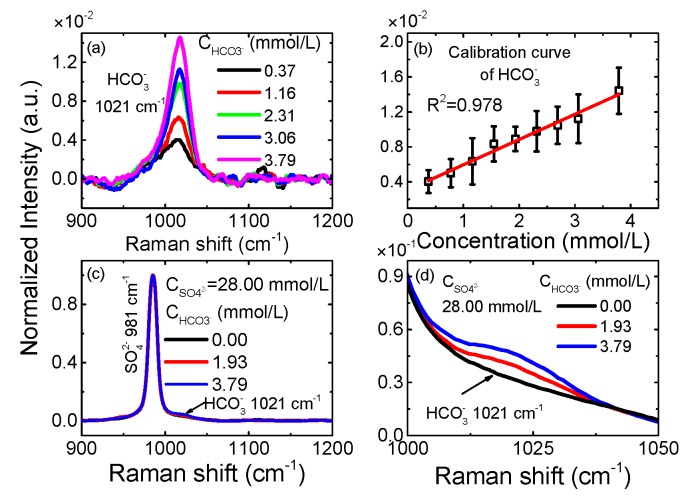
The detection ability of the NC-CERS system for HCO_3_^−^. (**a**) The spectra of 5 NaHCO_3_ aqueous solutions with different concentrations. (**b**) The linear relationship between concentrations and peak intensities of the HCO_3_^−^ signal. (**c**) The Raman spectra of three mixed solutions which contains fixed NaSO_4_ concentration (28.00 mmol/L) and different NaHCO_3_ concentrations. (**d**) The detailed spectra (1000–1050 cm^−1^) of mixed solutions.

To evaluate its performance for HCO_3_^−^, we prepared nine NaHCO_3_ solutions with concentrations ranging from 0.40 mmol/L to 4.00 mmol/L. The corresponding concentrations of HCO_3_^−^ are from 0.37 mmol/L to 3.79 mmol/L calculated by using the carbon balance mode. All of the concentrations of HCO_3_^−^ are corrected by using the equilibrium mode of carbon components in water. Figure 3a shows the spectra of five of these concentrations. The peak intensity of the HCO_3_^−^ Raman signal increases as a function of the concentration, and the Raman signal of HCO_3_^−^ can be distinguished in the solution with 0.37 mmol/L NaHCO_3_. It is proved that the LOD of the NC-CERS system for HCO_3_^−^ is less than 0.37 mmol/L, which is much lower than its concentration in seawater. The linear relationship between concentrations and peak intensities of the HCO_3_^−^ is shown in Figure 3b and it shows good linearity with R^2^ = 0.978. 

Furthermore, we prepared simulated seawater with fixed a NaSO_4_ concentration (28.00 mmol/L) and different NaHCO_3_ concentrations (0.00, 2.00, 4.00 mmol/L) to evaluate its ability to quantify HCO_3_^−^ in seawater. The corresponding concentrations of HCO_3_^−^ are 0.00, 1.93 and 3.79 mmol/L. The corresponding spectra are shown in Figure 3c, and the detailed spectra are shown in Figure 3d. Compared with the intensity of SO_4_^2−^, the intensity of the HCO_3_^−^ signal is much weaker. Even so, the Raman signal of HCO_3_^−^ can be detected in the spectrum of the mixed solution with 1.93 mmol/L NaHCO_3_. This shows the ability of the NC-CERS system for direct HCO_3_^−^ detection in seawater, but the strong and adjacent SO_4_^2−^ signal of seawater has an impact on the quantitative analysis of HCO_3_^−^.

**Figure 4:**
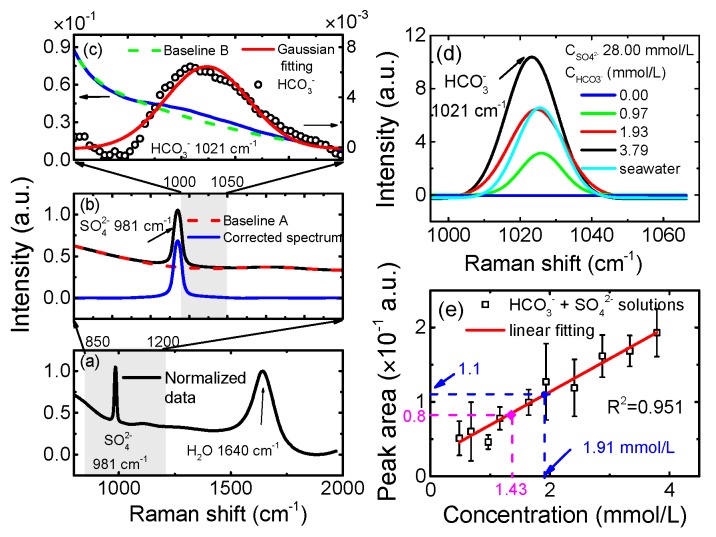
The signal extraction method for quantitative analysis of HCO_3_^−^. (**a**) The normalized spectrum of mixed solution with 28.00 mmol/L Na_2_SO_4_ and 1.93 mmol/L NaHCO_3_. (**b**) Baseline correction. The red dotted line represents the baseline curve fitted by a polynomial function, and the blue line is the baseline corrected curve. (**c**) The signal extraction method for HCO_3_^−^. The green dotted line represents the baseline B fitted by a double exponential function. The extracted signal is shown as black circle dots and fitted by using a Gaussian function (red line). (**d**) Fitted HCO_3_^−^ Raman signals of 1000 m depth seawater and four mixed solutions with fixed 28.00 mmol/L Na_2_SO_4_ and different concentrations of NaHCO_3_. (**e**) The calibration curve of HCO_3_^−^. The pink point is the result of a blind sample, and the blue point is the result of a seawater sample.

In order to reduce this impact, we developed an adaptive signal extraction method for HCO_3_^−^ and the process is shown in Figure 4a–c. The spectrum is obtained from a mixed solution with 28.00 mmol/L Na_2_SO_4_ and 1.93 mmol/L NaHCO_3_. In this spectrum, the Raman peaks of SO_4_^2−^ (981 cm^−1^) and H_2_O (1640 cm^−1^) can be clearly recognized, while the peak of HCO_3_^−^ (1021 cm^−1^) is almost submerged in the background. Given this, the original spectrum is normalized according to the peak intensity of H_2_O to reduce the impact of laser power fluctuation as shown in Figure 4a. The next step is baseline correction. We have compared four baseline fitting results with four methods (Voigt, Gaussian, Lorentzian and bi-exponential fitting methods), and the polynomial function shows the best performance among them. The baseline corrected result is shown as a blue line in Figure 4b. The last step is the weak HCO_3_^−^ signal extraction from the neighboring strong SO_4_^2−^ signal. The right wing of the SO_4_^2−^ signal is regarded as the baseline of the HCO_3_^−^ signal and is fitted by using a bi-exponential function in the region of 1000–1050 cm^−1^. We again tried four baseline fitting methods (Voigt, Gaussian, Lorentzian and bi-exponential fitting methods), and the polynomial function showed the best performance among them. The fitted baseline is shown as the green dotted line, and the corrected spectrum is shown as the black circle dots in Figure 4c. The Raman peak of HCO_3_^−^ is extracted and fitted by using a Gaussian function which is shown as the red line in Figure 4c.

Based on this data processing method, the Raman signals of HCO_3_^−^ in simulated seawater solutions are extracted and shown in Figure 4d. The signal of a deep seawater sample is also shown as the cyan line in Figure 4d. The linear relationship between concentrations and peak intensities of fitted HCO_3_^−^ is shown in Figure 4e and it shows high linearity with R^2^ = 0.951 over the range. We chose a mixed solution with 28.00 mmol/L NaSO_4_ and 1.50 mmol/L NaHCO_3_ as a blind sample. The test result is 1.35 mmol/L with 5.0% relative error, which is shown as the pink point in Figure 4e. Furthermore, we tested deep seawater, knowing the concentration of HCO_3_^−^ is 1.91 mmol/L which is shown as a blue point in Figure 4e. Compared with the reported HCO_3_^−^ concentration value of 1.95 mmol/L in seawater [4], the relative error was about 2.1%.

## 4. Conclusions

In this paper, we developed a near-concentric cavity-enhanced Raman spectroscopy system for liquid sample detection based on a near-concentric cavity and directly detected bicarbonate in seawater for the first time. Systematic experiments have been carried out for system evaluation. As shown by the NC-CERS system tests, the LOD for SO_4_^2−^ is less than 0.10 mmol/L, which is 15 times lower than the result reported before. The LOD for ${\mathrm{HCO}}_{3}^{–}$ is less than 0.37 mmol/L, which is much lower than its concentration in seawater (1.95 mmol/L). The detection performance of the NC-CERS system for HCO_3_^−^ in seawater is evaluated by analyzing mixed solutions of fixed 28.00 mmol/L NaSO_4_ and different NaHCO_3_ concentrations. The Raman signal of 1.93 mmol/L HCO_3_^−^ in mixed solution can be clearly detected, which shows the ability of direct HCO_3_^−^ detection in seawater. To further realize the quantitative analysis for HCO_3_^−^, a specially developed data processing scheme is introduced. With this data processing method, the weak HCO_3_^−^ signal is extracted from the neighboring strong SO_4_^2−^ signal, hence a quantitative analysis with R^2^ = 0.951 is made possible. Based on this calibration curve, the HCO_3_^−^ concentrations of a blind sample and a seawater sample are calculated. The calculated values are 1.43 mmol/L and 1.91 mmol/L with 4.9% and 2.1% relative error, respectively. It is hoped that this NC-CERS system could be fully developed for field ocean observations in the near future. CO_3_^2−^ with a concentration down to 1/10 of the HCO_3_^−^ in seawater will be the next detection object.
